# Ocular Hypertension: General Characteristics and Estimated Cerebrospinal Fluid Pressure. The Beijing Eye Study 2011

**DOI:** 10.1371/journal.pone.0100533

**Published:** 2014-07-02

**Authors:** Jost B. Jonas, Ningli Wang, Ya Xing Wang, Qi Sheng You, Diya Yang, Liang Xu

**Affiliations:** 1 Beijing Institute of Ophthalmology, Beijing Tongren Eye Center, Beijing Tongren Hospital, Capital Medical University, Beijing Ophthalmology and Visual Science Key Lab, Beijing, China; 2 Department of Ophthalmology, Medical Faculty Mannheim of the Ruprecht-Karls-University of Heidelberg, Mannheim, Germany; 3 Beijing Tongren Eye Center, Beijing Tongren Hospital, Capital Medical University, Beijing Ophthalmology and Visual Sciences Key Laboratory, Beijing, China; Casey Eye Institute, United States of America

## Abstract

**Purpose:**

To examine characteristics of ocular hypertensive subjects and potential associations with estimated cerebrospinal fluid pressure (estCSFP).

**Methods:**

The population-based Beijing Eye Study 2011 included 3468 individuals with a mean age of 64.6±9.8 years. Ocular hypertension was defined as intraocular pressure (IOP) >21 mmHg, normal optic nerve head appearance and normal retinal nerve fiber layer thickness. IOP was corrected for its dependence on central corneal thickness (CCT) and corneal curvature radius. Estimated CSFP was calculated as CSFP [mmHg] = 0.44×Body Mass Index [kg/m^2^]+0.16×Diastolic Blood Pressure [mmHg]−0.18×Age [Years]−1.91. Estimated trans-lamina cribrosa pressure difference (estTLCPD) was IOP–estCSFP.

**Results:**

EstCSFP (10.5±3.6 mmHg versus 9.0±3.7 mmHg; P = 0.003) and estTLCPD (12.0±4.4 mmHg versus 5.4±3.8 mmHg; *P*<0.001) were higher in the ocular hypertensive group than in the normotensive group. In binary regression analysis, ocular hypertension was associated with increased estCSFP (*P* = 0.03; odds ratio (OR): 1.08; 95% confidence interval (CI): 1.01, 1.17) after adjusting for prevalence of arterial hypertension (*P* = 0.07; OR: 1.79; 95%CI: 0.96, 3.34), retinal nerve fiber layer thickness (*P* = 0.03; OR: 0.97; 95%CI: 0.95, 0.997) and blood glucose concentration (*P* = 0.006; OR: 1.17; 95%CI: 1.04, 1.30).

**Conclusions:**

Ocular hypertensive subjects (with IOP correction for CCT and corneal curvature) as compared to ocular normotensive subjects had a significantly higher estCSFP in univariate analysis and in multivariate analysis. Despite of a higher estCSFP, estTLCPD was still markedly higher in ocular hypertensive eyes than in ocular normotensive eyes.

## Introduction

Ocular hypertension has been defined by an intraocular pressure (IOP) higher than 21 mm Hg, a normal optic nerve and normal visual field [1]. It has been considered to be the opposite of normal-pressure glaucoma in which despite a normal IOP progressive glaucomatous optic neuropathy develops [2]. Several reasons for the clinical spectrum spanning from ocular hypertension to normal-pressure glaucoma have been discussed including inter-individual differences in IOP-related glaucoma susceptibility, differences in the level and influence of arterial blood pressure on the optic nerve head, vasospastic factors and others [2], [3]. Recent studies have shown a physiologic correlation between IOP, blood pressure and cerebrospinal fluid pressure (CSFP) [4]. Subjects with higher IOP had higher arterial blood pressure and higher CSFP, and vice versa. These findings and the anatomy of the optic nerve head as pressure barrier between the intraocular compartment with the IOP and the retrobulbar compartment with the CSFP led to the hypothesis, that ocular hypertensive subjects may have a relatively high CSFP, and vice versa that a low CSFP may be associated with the pathogenesis of normal-pressure glaucoma [4]–[13]. The higher CSFP in ocular hypertensive subjects would counterbalance against an increased IOP so that the trans-lamina cribrosa pressure difference (TLCPD) would be in the normal range. We tested this hypothesis in a population-based study in which the CSFP was estimated based on diastolic blood pressure, body mass index and age [14], [15]. Previous investigations had revealed that these three parameters markedly determine the CSFP in neurologically mostly normal subjects [14], [16], [17]. We chose a population-based study design to avoid a referral induced bias in the selection of study participants.

## Methods

### Ethics Statement

The Medical Ethics Committee of the Beijing Tongren Hospital approved the study protocol and all participants gave informed written consent, according to the Declaration of Helsinki.

The Beijing Eye Study 2011 is a population-based cross-sectional study in Northern China on subjects aged 50+ years [18], [19]. Out of an eligible population of 4403 individuals, 3468 (78.8%) individuals participated with a mean age of 64.6±9.8 years (median, 64 years; range, 50–93 years). All study participants underwent an interview with standardized questions on their family status, level of education, income, quality of life, physical activity, known major systemic diseases such as arterial hypertension and diabetes mellitus, and quality of vision. Fasting blood samples were taken for measurement of blood lipids, glucose and glycosylated hemoglobin HbA1c. Cognitive function was assessed using the MMSE (mini mental state examination) scale [20]. Body height and weight and blood pressure were measured in a standardized manner [21]. Arterial hypertension was defined as a systolic blood pressure ≥140 mm Hg and/or a diastolic blood pressure ≥90 mm Hg, and/or self-reported current treatment for arterial hypertension with antihypertensive medication. The study design and the techniques have been described in detail recently [18], [19], [21]. The ophthalmic examination included measurement of visual acuity, tonometry, slit lamp examination of the anterior and posterior segment of the eye, ocular biometry (Lensstar 900 Optical Biometer, Haag-Streit, 3098 Koeniz, Switzerland), and digital photography of the cornea, lens, macula and optic disc (fundus camera Type CR6-45NM; Canon Inc, Tokyo, Japan). Using the optic disc photographs, the width of the neuroretinal rim and the diameters of the optic cup and optic disc were measured in the vertical meridian of the optic disc [21]. The vertical cup/disc diameter ratio (VCDR) was calculated. Additionally, we performed a spectral-domain optical coherence tomography (SD-OCT) for measurement of the retinal nerve fiber layer and choroidal thickness (Wavelength: 870 nm; Heidelberg Engineering Co., Heidelberg, Germany) in all study participants as well as a SD-OCT for measurements of the optic nerve head (iTVue SD-OCT; Optovue Inc. Fremont, CA, U.S.A.) in a randomized subgroup of 1654 study participants.

The measured IOP was corrected for its dependence on central corneal thickness (CCT) and anterior corneal curvature radius [22], [23]. In our study population, IOP was significantly associated with thinner central corneal thickness (*P*<0.001) and shorter corneal curvature radius (*P*<0.001) after adjusting for parameters such shallower anterior chamber depth, longer axial length, younger age, higher pulse rate, higher prevalence of arterial hypertension, and higher blood concentration of triglycerides and cholesterol. The regression coefficients of the associations between IOP and CCT or corneal curvature were used for the formula of the corrected IOP: “IOP_corrected_ = IOP_measured_−((CCT [µm]−532)×0.038)+((Corneal Curvature Radius [mm]−7.62)×1.11)”. Mean CCT was 532 µm, and mean corneal curvature radius was 7.62 mm. Using this formula to arrive at the corrected IOP, the measured IOP was decreased by 3.8 points for each 100 µm which the cornea was thicker than the average mean of 538 µm, and the measured IOP was increased by 1.11 points for each mm, the corneal curvature radius was larger than the average mean of 7.62 mm. This formula was partially similar to the one developed by Kohlhaas and colleagues, in which an approximately 1-mm Hg correction for every 25-µm deviation from a CCT of 550 µm was suggested [24]. In our formula, the correction for each 25 µm difference from the mean CCT (of 532 µm) was 0.95 mm Hg. The difference between Kohlhaas' formula and the formula applied in the present study was that in Kohlhaas' formula the dependence of IOP measurements on corneal curvature was not taken into account and that it was based on Caucasian eyes in which as compared to Chinese eyes the mean CCT is thicker.

Ocular hypertension was defined by a corrected IOP higher than 21 mm Hg, a normal retinal nerve fiber layer as measured by spectral-domain OCT, and a normal appearance of the optic nerve head. The latter was defined by a normal shape of the neuroretinal rim following the ISNT (Inferior-Superior-Nasal-Temporal) rule [25]. The assessment of the optic disc photographs was carried in a masked manner. Each optic disc photograph of an ocular hypertensive subject was independently adjudicated by three senior graders (LX, YXW, JBJ).

**Figure 1:**
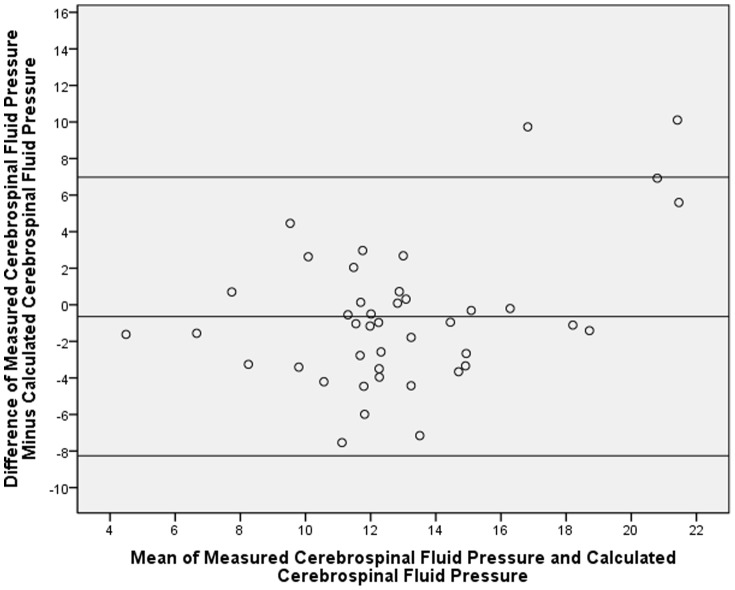
Bland-Altman Plot Showing the Distribution of the Mean of the Calculated Cerebrospinal Fluid Pressure (CSFP) and Measured CSFP Versus the Mean Difference of Measured CSFP – Calculated CSFP.

For the calculation of a formula to estimate the CSFP, we used the lumbar CSFP measurements obtained in a previous pilot study [14]. This was a prospective observational comparative study on patients who consecutively underwent lumbar puncture for diagnosis and treatment of neurological diseases. The lumbar CSFP was measured in a standardized manner at 14:00 hours. The study included 72 patients with a mean age of 42.0±13.4 years. The mean CSFP was 12.6±4.8 mm Hg. The final diagnosis of the patients included diseases such as peripheral neuropathy, multiple sclerosis, unilateral ischemic optic neuropathy and unilateral optic neuritis, in which it was unlikely that the neurological disease was associated with an abnormal CSFP. Out of the total group, we randomly formed a training group consisting of 32 patients, and a testing group including the remaining 42 patients. Due to randomization, the training group and testing group did not differ significantly in age, gender, body height and weight, body mass index, intraocular pressure, retinal nerve fiber layer thickness, and arterial blood pressure (all *P*>0.10). Performing a multivariate analysis in the training group with the lumbar CSFP measurements as dependent variable and age, body mass index and blood pressure as independent variables revealed, that estimated CSFP was best described by the formula of Estimated CSFP [mmHg] = 0.44×Body Mass Index [kg/m^2^]+0.16×Diastolic Blood Pressure [mmHg]−0.18×Age [Years]−1.91 [14], [15]. The correlation coefficient was r = 0.55. The association between higher CSFP and younger age, higher body mass index and higher blood pressure had also been found in other previous investigations [16], [17]. We then tested the formula in the testing group. In this testing group, the measured lumbar CSFP (12.6±4.8 mm Hg) did not differ significantly (*P* = 0.29) from the calculated CSFP (13.3±3.2 mm Hg). The coefficient for the correlation between the calculated CSFP value and the measured lumbar CSFP was r: 0.59. The Durbin-Watson value was 2.08. Durbin-Watson values falling into the acceptable range of 1.5 to 2.5 indicate a non-significant autocorrelation for the residuals in the multiple regression models. The intra-class correlation coefficient was 0.71. The Bland-Altman analysis revealed that 40 out of 42 measurements were within the 95% limits of agreement (Fig. 1).

Inclusion criteria for the present study were the availability of assessable optic disc photographs, OCT images for the assessment of retinal nerve fiber layer thickness measurements, measurements of IOP, central corneal thickness and anterior corneal curvature radius, body mass index values, and diastolic blood pressure measurements.

Statistical analysis was performed using a commercially available statistical software package (SPSS for Windows, version 21.0, IBM-SPSS, Chicago, IL). First, we examined the mean values (presented as mean ± standard deviation). Second, we corrected the IOP measurements for the dependence on CCT and corneal curvature as described above. Third we identified the ocular hypertensive group and looked for differences between the later and the ocular normotensive group in univariate analysis. Fourth, we performed a multivariate binary regression analysis with the presence of ocular hypertension as dependent variable and all parameters as independent variables which were significantly associated with ocular hypertension in the univariate analysis. We then dropped those parameters which were no longer significantly associated with ocular hypertension. Fifth, we searched for associations between the estimated CSFP and other parameters including IOP. Only one randomly selected eye per individual was included into the statistical analysis. Odds ratios (OR) 95% confidence intervals (CI) were presented. All *P*-values were 2-sided and were considered statistically significant when the values were smaller than 0.05.

## Results

Measurements of IOP, central corneal thickness, corneal curvature radius, blood pressure, and body height and weight and assessable optic disc photographs and OCT images were available for 6064 (87.4%) eyes. Out of these, 5280 eyes of 2819 subjects (1607 (57.0%) women) fulfilled the inclusion criteria of a normal retinal nerve fiber layer and a normal appearance of the optic nerve head. Mean age was 63.7±9.4 years (median: 62 years; range: 50 to 93 years) and mean refractive error was −0.03±1.70 diopters (median: 0.25 diopters; range: −15.0 to +7.00 diopters).

**Figure 2:**
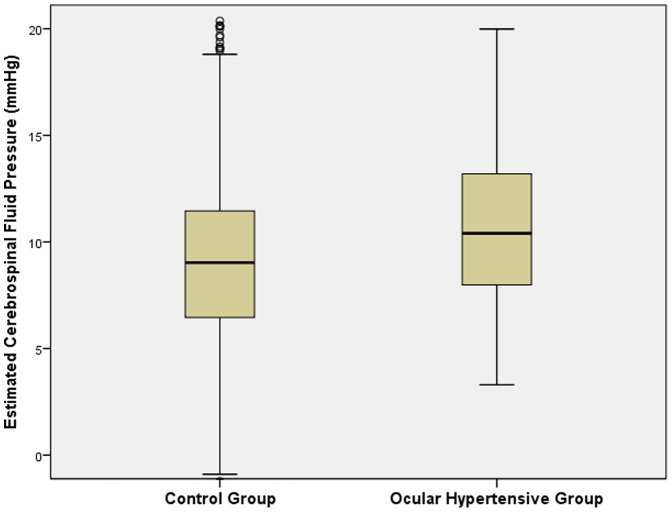
Boxplot showing the distribution of the estimated cerebrospinal fluid pressure in the ocular hypertensive subjects and normal subjects in the Beijing Eyes Study 2011.

**Figure 3:**
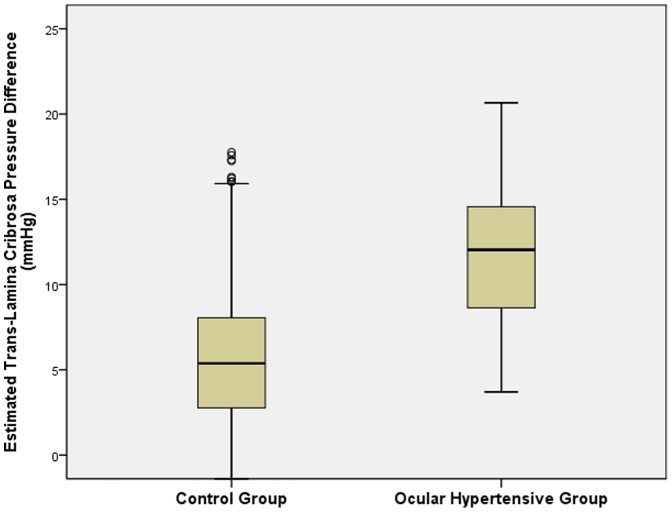
Boxplot showing the distribution of the estimated trans-lamina cribrosa pressure difference in the ocular hypertensive subjects and normal subjects in the Beijing Eyes Study 2011.

Mean IOP was 14.7±2.7 mm Hg (median: 14.0 mmHg; range: 5–37 mm Hg) and mean corrected IOP was 14.7±2.5 mm Hg (median: 14.5 mm Hg; range: 6.6–35.6 mm Hg). The definition of ocular hypertension was fulfilled by 70 (1.3%) eyes of 62 (2.2%) subjects (32 (52%) women). For 51 of the 70 ocular hypertensive eyes, results of frequency doubling perimetry performed at the baseline survey in 2001 were available. All test points were tested normal in 48 (94%) eyes, while one (2%) eye had one test point positive. Two additional eyes (4%) showed an unreliable visual field test result. Mean age of the ocular hypertensive subjects was 63.6±8.9 years (range: 50–84 years) and mean refractive error was 0.16±1.66 diopters (range: −5.50 to +6.38 diopters). In the ocular hypertensive group as compared with the ocular normotensive group, estimated CSFP (10.4±3.7 mm Hg versus 9.1±3.7 mm Hg; *P* = 0.003) and estimated TLCPD (12.1±4.5 mm Hg versus 5.5±3.8 mm Hg; *P*<0.001) were significantly higher (Table 1) (Fig. 2, 3). Arterial hypertension was present in 1490 (52.9%) individuals of the study population out of whom 1123 (75.4%) subjects were on antihypertensive therapy.

**Table 1:** Demographic Data and Measurements (Mean ± Standard Deviation) of Ocular Hypertensive Subjects and Ocular Normotensive Subjects with Normal Optic Nerve in the Beijing Eye Study 2011.

Parameter	Ocular Hypertensive Group	Normotensive Group	*P*-Value
N	62	2757	
Age (Years)	63.3±8.8	63.7±9.5	0.73
Rural Region/Urban region	42/20	1327/1430	0.003
Men/Women	30/32	1182/1575	0.44
Body Mass Index (kg/m^2^)	26.6±4.2	25.6±3.8	0.07
Body Height (cm)	162±9	162±8	0.90
Blood Concentration Glucose (mmol/L)	6.41±2.49	5.69±1.52	0.04
Glycosylated Hemoglobin (%)	4.69±1.48	4.36±0.95	0.10
Blood Concentration Cholesterol (mmol/L)	5.26±1.24	5.08±1.02	0.30
Blood Concentration High-Density Lipoproteins (mmol/L)	1.41±0.50	1.45±0.46	0.59
Blood Concentration Low-Density Lipoproteins (mmol/L)	3.52±1.06	3.37±0.92	0.32
Blood Concentration Triglycerides (mmol/L)	2.24±2.09	1.75±2.64	0.10
Systolic Blood Pressure (mm Hg)	140±23	130±21	0.001
Diastolic Blood Pressure (mm Hg)	76±12	70±12	0.001
Pulse	74.8±11.8	72.3±10.0	0.10
Arterial Hypertension (%)	72.6±5.7	52.4±1.0	0.002
Treatment of Arterial Hypertension	52.5±6.4	40.5±0.9	0.07
Diabetes mellitus (%)	23.6±5.8	19.2±0.8	0.39
Cognitive Function Score	26.2±3.6	26.5±3.	0.44
Estimated Cerebrospinal Fluid Pressure (mm Hg)	10.5±3.6	9.0±3.7	0.003
Intraocular Pressure (Corrected) (mm Hg)	22.5±2.1	14.4±2.3	<0.001
Trans-Lamina Cribrosa Pressure Difference (Corrected) (mm Hg)	12.0±4.4	5.4±3.8	<0.001
Retinal Nerve Fiber Layer Thickness (µm)	100±9	103±10	0.02
Refractive Error (Diopter)	0.23±1.59	−0.04±1.70	0.48
Central Corneal Thickness (µm)	532±39	532±32	0.99
Anterior Corneal Curvature Radius (mm)	7.62±0.26	7.61±0.25	0.87
Anterior Chamber Depth (mm)	2.33±0.35	2.48±0.46	0.30
Lens Thickness (mm)	4.64±0.27	4.56±0.33	0.12
Axial Length (mm)	22.9±1.2	23.2±0.97	0.29

In univariate analysis, the ocular hypertensive group as compared with the normotensive group had a significantly higher systolic and diastolic blood pressure (*P* = 0.001), higher prevalence of the rural region as site of habitation (*P* = 0.003), higher values for the blood concentration of glucose (*P* = 0.04), higher estimated CSFP (*P* = 0.003) and higher estimated TLCPD (*P*<0.001), and thicker retinal nerve fiber layer (*P* = 0.02) (Table 1). Due to the selection, IOP was significantly higher in the ocular hypertensive group. The ocular hypertensive group and the normotensive group did not differ significantly in the systemic parameters of age, gender, body mass index, body height, pulse rate, prevalence of diabetes mellitus, and cognitive function score, and the ocular biometric parameters of central corneal thickness, anterior chamber depth, lens thickness and axial length, and refractive error.

In the binary regression analysis, ocular hypertension remained significantly associated with increased estimated CSFP (*P* = 0.02), thinner retinal nerve fiber layer (*P* = 0.03), higher blood concentration of glucose (*P* = 0.006), and marginally significantly with higher prevalence of arterial hypertension (*P* = 0.07) (Table 2). If treatment of arterial hypertension was added to the list of independent variables in the multivariate model, it was not significantly (*P* = 0.71; OR: 1.04; 95%CI: 0.84, 1.30) correlated with ocular hypertension. If due to its statically marginal association with ocular hypertension arterial hypertension was dropped from the model, the association between ocular hypertension and estimated CSFP became stronger (*P* = 0.01; OR: 1.10; 95%CI: 1.02, 1.19).

**Table 2:** Multivariate Analysis of the Associations between the Presence of Ocular Hypertension and Systemic and Ocular Parameters in the Beijing Eye Study 2011.

Parameter	*P*-Value	Regression Coefficient B	Odds Ratio	95% Confidence Interval
Estimated Cerebrospinal Fluid Pressure (mm Hg)	0.03	0.08	1.08	1.01, 1.17
Arterial Hypertension	0.07	0.58	1.79	0.96, 3.34
Retinal Nerve Fiber Layer Thickness (µm)	0.03	−0.03	0.97	0.95, 0.997
Blood Concentration Glucose (mmol/L)	0.006	0.15	1.17	1.04, 1.30

## Discussion

In our population-based study with correction of the IOP measurements for their dependence on CCT and corneal curvature radius, ocular hypertensive subjects had a significantly higher estimated CSFP after adjusting for arterial hypertension, blood glucose concentration and retinal nerve fiber layer thickness. Despite of the higher estimated CSFP, however, the estimated TLCPD was still markedly higher in ocular hypertensive eyes than in ocular normotensive eyes.

These results confirm a previous hospital-based study by Ren and colleagues [12], and they indirectly fit with the findings of other previous studies which also addressed the estimated TLCPD as compared to the IOP as the main pressure-related parameter for the optic nerve head [4], [5], [7], [9]–[14], [26]–[28]. The TLCPD was defined as the difference between IOP minus CSFP, while the IOP, in a strict physical sense, is just the transcorneal pressure difference between the intraocular compartment and the surrounding external atmosphere. Since the lamina cribrosa of the optic nerve head, as the presumed site of glaucomatous damage to the optic nerve, is the pressure shed between the intraocular compartment and the retrolaminar compartment with the cerebrospinal fluid space, it has been discussed that the TLCPD as compared with the IOP may be more important for the pathophysiology of the optic nerve head including the development of glaucomatous optic neuropathy. That notion was supported by experimental, clinical and anatomical investigations. Anatomical investigations discussed that the orbital CSFP is the trans-laminar cribrosa counter-pressure against the IOP, since the cerebrospinal fluid space extends from the intracranial compartment along the optic nerve as fascicle of the brain into the orbit and ends at the peripapillary scleral flange of the optic nerve head [6], [8]. Clinical studies suggested that some patients with glaucomatous optic nerve damage and normal IOP may have an abnormally low CSFP [4], [9]–[12]. The results and conclusions of these studies were supported by observations made in a recent investigation in which the width of the orbital CSF space was significantly smaller in patients with normal-pressure glaucoma than in patients with high-pressure glaucoma or in normal subjects [13]. The orbital CSF space width depends on the intracranial CSFP [14]. In a recent analysis of the Central India eye and Medical Study, presence of open-angle glaucoma was significantly associated with estimated TLCPD but not with IOP in multivariate analysis, while the prevalence of angle-closure glaucoma was significantly associated with IOP but not with estimated TLCPD [15]. In open-angle glaucoma but not in angle-closure glaucoma, estimated TLCPD versus IOP showed a better association with glaucoma presence and amount of glaucomatous optic neuropathy

In the population-based Central India Eye and Medical Study and in the Beijing Eye Study, a smaller neuroretinal rim which can be considered as surrogate for glaucomatous optic nerve damage, was associated with a lower body mass index [29], [30], which in other studies was associated with a low CSFP as measured by direct lumbar puncture [16], [17]. It suggested that a high body mass index was protective against a small neuroretinal rim. Due to the association between CSFP and body mass index, the body mass index is part of the formula to estimate the CSFP. The finding of the Central India Eye and Medical Study thus fits with the results of our present investigation.

The results of our study may suggest that the elevated IOP in eyes with ocular hypertension is partially compensated by an elevated CSFP, so that the TLCPD gets relatively reduced. Interestingly, however, the estimated TLCPD was, despite of the slightly higher CSFP, still markedly higher in the ocular hypertensive group than in the control group (12.1±4.5 mm Hg versus 5.5±3.8 mm Hg; *P*<0.001) (Table 1). One may therefore wonder whether besides an elevated CSFP, other additional parameters may be at play in eyes which did not develop glaucomatous optic neuropathy despite elevated IOP. One potentially important aspect may be the dynamics of the pressure changes. Morgan and colleagues demonstrated a difference in the phasing of the IOP curve and the phasing of the CSFP curve with respect to the cardiac cycle, with the intracranial pressure curve reaching its height earlier than the IOP curve [31]. The time difference between the pressure waves on both sides of the lamina cribrosa may lead to pulse-synchronous changes in the TLCPD. It may even be wondered whether for a fraction of a second, the orbital CSFP may be higher than the IOP, leading to a reversal of the TLCPD [32]. This swinging of TLCPD may potentially be necessary to allow the retrograde axoplasmic flow entering the eye.

In previous population-based studies, such as the Rotterdam Study and the Tanjong Pagar Study [33], [34], higher IOP was associated with higher blood pressure, higher pulse rate and/or higher prevalence of arterial hypertension. These findings are confirmed by the results of our investigation in which the ocular hypertensive group as compared with the normotensive group had a significantly (*P*<0.001) higher systolic and diastolic blood pressure (Table 1).

The findings obtained in our present study fit with observations made in another recent study. In the Central India Eye and Medical Study, the mean estimated TLCPD was 3.6±4.3 mm Hg in the non-glaucomatous population and 9.67±8.2 mm Hg in the glaucomatous group [15]. In multivariate analysis, higher estimated TLCPD was associated with the presence of glaucomatous optic neuropathy (*P*<0.001; beta:0.11; B:3.43; 95%CI:2.96,3.99). Differences between glaucomatous subjects and non-glaucomatous subjects in estimated CSFP were more pronounced for open-angle glaucoma than for angle-closure glaucoma (3.0 mmHg versus 1.8 mmHg), while differences between glaucomatous subjects and non-glaucomatous subjects in IOP were higher for angle-closure glaucoma than for open-angle glaucoma (8.5 mmHg versus 3.0 mmHg). Presence of open-angle glaucoma was significantly associated with estimated TLCPD (*P*<0.001; OR:1.24; 95%CI:1.19,1.29) but not with IOP (*P* = 0.08; OR:0.96; 95%CI:0.91,1.00). Prevalence of angle-closure glaucoma was significantly associated with IOP (*P = *0.04; OR:1.19; 95%CI:1.01,1.40) but not with estimated TLCPD (*P* = 0.92). It was concluded that in open-angle glaucoma, but not in angle-closure glaucoma, estimated TLCPD versus IOP showed a better association with glaucoma presence and amount of glaucomatous optic neuropathy. Similar results were obtained in the Beijing Eye Study, in which IOP was higher (*P* = 0.008), estimated CSFP was lower (*P*<0.001) and estimated TLCPD was (*P*<0.001) higher in the glaucoma group than in the non-glaucomatous group [35]. The inter-group difference was highest for estimated TLCPD followed by estimated CSFP and finally IOP. As in the Central India Eye and Medical Study, open-angle glaucoma was associated with higher estimated TLCPD (*P*<0.001) but not with IOP (*P* = 0.22), while angle-closure glaucoma was associated with higher IOP (*P* = 0.03) but not with estimated TLCPD (*P* = 0.98) in multivariate analysis. In the Central India Eye and Medical Study as well as in the Beijing Eye Study, higher estimated CSFP was correlated with higher IOP in univariate analysis and in multivariate analysis [15]. One of the reasons for this association may have been that both CSFP and IOP are positively correlated with body mass index [16], [17], [36].

Interestingly, recent studies revealed that the estimated CSFP was correlated with other ocular parameters and diseases, in addition to ocular hypertension and open-angle glaucoma. A higher estimated CSFP was associated with a thicker subfoveal choroidal thickness, wider retinal veins, in particular in arterial hypertensive patients, a higher intraocular pressure (own data) and a higher prevalence of diabetic retinopathy [37]–[39].

Potential limitations of our study should be mentioned. First, the whole statistical analysis depended on the formula to calculate the CSFP. The study, in which the basis parameter for that formula were assessed included a relatively small number of subjects, and these subjects had a clinic al reason to undergo lumbar puncture [14]. Although the clinical neurological examination and the further clinical course retrospectively revealed that it was unlikely that the lumbar CSFP measurement in that study group was markedly influenced by the reason to perform the lumbar puncture, one has to keep in mind, that the participants in that study were not randomly selected normal subjects. One may also consider that the correlation coefficient for the association between calculated CSFP and measured CSFP was relatively low, however, it was higher in the testing group (r = 0.59) than in the training group (r = 0.55). Also, the Bland-Altman plot showed a considerable scattering, indicating a relative impreciseness of the formula (Fig. 1). Without doubt, future studies have to corroborate the formula to estimate the CSFP in normal subjects. Second, perimetry was not performed so that the criterion of a normal visual field for the common definition of ocular definition could not be assessed. The optic nerve head appearance as assessed in a masked manner by a panel of three senior graders (LX, YXW, JBJ) was however normal and the retinal nerve fiber layer as measured by OCT was unremarkable. It may make it unlikely, that more than few eyes included into the group of ocular hypertension had (early) visual field defects, since changes in the optic nerve head and retinal nerve fiber layer usually precede perimetric defects in glaucoma [40]. Third, IOP was measured only once, so that the question arises how representative this single IOP measurement was for the subject's IOP in general. Although the single IOP measurements may have increased the noise (or decreased representativeness) of the measurements, the associations in the multivariate analysis were statistically significant, so that this limitation in the study design may serve to strengthen the conclusions of the study. Fourth, it must be considered that the coefficients for the relationships between ocular hypertension and some other parameters were relatively low (Table 2). Although these relationships were statistically significant with a *P*-value of <0.05, the low coefficients showed that only a fraction of the variability in the prevalence of ocular hypertension could be explained by that relationship. Fifth, as for any population-based study, the rate of non-participation or non-availability of examination results can matter. In our study, the participation rate was 78.8% what may be acceptable.

In conclusion, ocular hypertensive subjects as compared to ocular normotensive subjects had a significantly higher estimated CSFP after adjusting for arterial hypertension, pulse rate, blood glucose concentration and retinal nerve fiber layer thickness. Correspondingly, higher CSFP was associated with higher IOP. Despite of the higher estimated CSFP, the estimated TLCPD was markedly higher in ocular hypertensive eyes than in ocular normotensive eyes. Besides an elevated CSFP, other additional parameters may be at play in eyes which did not develop glaucomatous optic neuropathy despite elevated IOP.
